# Asymmetrical evolution of cross inhibition in zooplankton: insights from contrasting phosphorus limitation and salinization exposure sequences

**DOI:** 10.1098/rspb.2024.3064

**Published:** 2025-03-05

**Authors:** Libin Zhou, Kimberley D. Lemmen, Shuaiying Zhao, Steven A. J. Declerck

**Affiliations:** ^1^ Key Laboratory of Lake and Watershed Science for Water Security, Nanjing Institute of Geography and Limnology, Chinese Academy of Sciences, Nanjing, People’s Republic of China; ^2^ State Key Laboratory of Lake Science and Environment, Nanjing Institute of Geography and Limnology, Chinese Academy of Sciences, Nanjing, People’s Republic of China; ^3^ Department of Aquatic Ecology, Netherlands Institute of Ecology (NIOO-KNAW), Wageningen, The Netherlands; ^4^ Department of Evolutionary Biology and Environmental Studies, University of Zurich, Zurich, Switzerland; ^5^ ISEM, University of Montpellier, CNRS, IRD, Montpellier, France; ^6^ Yunnan Key Laboratory of Plateau Geographical Processes and Environmental Change, Faculty of Geography, Yunnan Normal University, Kunming, People’s Republic of China; ^7^ Laboratory of Aquatic Ecology, Evolution and Conservation, Department of Biology, KU Leuven, Leuven, Belgium

**Keywords:** rapid adaptation, phosphorus limitation, salinization, cross inhibition, cross-tolerance, rotifer

## Abstract

Understanding the evolutionary responses of organisms to multiple stressors is crucial for predicting the ecological consequences of intensified anthropogenic activities. While previous studies have documented the effects of selection history on organisms' abilities to cope with new stressors, the impact of the sequence in which stressors occur on evolutionary outcomes remains less understood. In this study, we examined the evolutionary responses of a metazoan rotifer species to two prevalent freshwater stressors: nutrient limitation and increased salinization. We subjected rotifer populations with distinct selection histories (salt-adapted, low phosphorus-adapted and ancestral clones) to a reciprocal common garden experiment and monitored their population growth rates. Our results revealed an asymmetric evolutionary response to phosphorus (P) limitation and increased salinity. Specifically, adaptation to low P conditions reduced rotifer tolerance to increased salinity, whereas adaptation to saline conditions did not show such cross-inhibitory effects. Instead, the addition of moderate concentrations of salt enhanced the growth of the salt-adapted population in low P conditions, potentially as a consequence of evolved cross-tolerance. Our findings, therefore, underscore the importance of considering historical stressor regimes to improve our understanding and predictions of organismal responses to multiple stressors and also have significant implications for ecosystem management.

## 1. Introduction

The Anthropocene is characterized by the emergence of novel stressors, accompanied by the intensification of a broad spectrum of naturally occurring stressors affecting individual organisms, populations and communities of natural ecosystems [1,2]. The question of how these stressors interact and impact organisms has become a major research topic in ecology [3–5]. Interactive effects of multiple stressors can be intricate, and their outcome does not only depend on stressor type and organismal traits but also on the environmental context and temporal patterns of exposure [1,6,7].

While most research has focused on how simultaneous stressor interactions affect organisms [6,8,9], the temporal dynamics of stressors, particularly the sequence and duration of their overlap, have recently emerged as crucial yet understudied factors in shaping ecological outcomes [4]. Evidence for the significance of stressor sequence comes mainly from physiological studies. For example, prior exposure to elevated temperature enhances both heat and salinity tolerance in *Daphnia pulex*, while increased salinity at low temperatures reduces their capacity to tolerate heat and salinity [10]. Similarly, Ashauer *et al*. [11] found that the effects of toxicants on freshwater *Gammarus pulex* varied depending on the sequence of exposure. Moreover, theoretical studies have suggested that exposure to contaminants can enhance organisms' susceptibility to infectious diseases by compromising their immune system [12]. This increased vulnerability may be particularly influenced by the sequence of exposure to these stressors [13]. These examples highlight the importance of considering the temporal dynamics, and more specifically the importance of sequence of multiple stressors in natural systems.

Rapid microevolutionary adaptation to stressors is increasingly recognized as having potentially profound impacts on ecological dynamics [14]. A recent meta-analysis of eco-evolution studies suggests that in a majority of cases, such adaptation dampens the effect of ecological change [15]. An interesting question that emerges is how a history of adaptation to one stressor may affect its ability to cope with another stressor. Cross-inhibition evolves when adaptation to one stressor may lead to a reduced ability to mitigate other stressors [16–19], a phenomenon that often originates from trade-offs among functional traits in organisms [20,21], including antagonistic pleiotropy [22,23]. Conversely, cross-tolerance evolves when adaptation to one stressor enhances an organism’s ability to tolerate other stressors [24–28]. This phenomenon arises when selection under a specific stressor induces adaptive responses that subsequently confer tolerance to other stressors [29]. Despite significant advances in our understanding of the consequences of adaptation to specific stressors, we still lack a clear understanding of how the order in which stressors are encountered influences an organism’s ability to cope with them. When considering two stressors, a ‘symmetric’ evolutionary response occurs when adaptation to either stressor has a similar effect on tolerance, either positive or negative, to the alternative stressor. Alternatively, adaptation to an initial stressor may differently affect an organism’s tolerance to a second stressor depending on the sequence in which they are experienced. More specifically, adaptation to one stressor may enhance an organism’s ability to handle a subsequent stressor, while adaptation to the second stressor may reduce the organism’s tolerance to the first, or vice versa (‘asymmetric’ response). For example, in a study with *Saccharomyces cerevisiae*, prior exposure to oxidative stress enhanced the yeast’s resistance to salt stress, but the reverse sequence did not confer cross-protection [30]. Such varying consequences of adaptation could have important implications for the fate of natural populations, including their ability to persist under complex and temporally varying multi-stressor regimes. Furthermore, they could have cascading effects on species interactions, biodiversity and ecosystem functioning, highlighting the need for a better understanding of these processes to predict how ecosystems will respond to future stressor regimes.

In the era of the Anthropocene, freshwater systems are increasingly being threatened by a variety of stressors. For example, salinization has recently emerged as an issue of global concern [31–33] due to climate change-driven alterations in global evaporation–precipitation budgets [34–36], and direct human impacts such as road salt application and industrial discharge [32]. Increased salinity levels compromise freshwater organisms through ion toxicity and osmotic stress [29,37–39], and undermine food web integrity and ecosystem function [40]. For example, elevated salinity may impose energetic constraints on zooplankton predator avoidance behaviours and vertical migration, and consequently make them more vulnerable to predation, reducing their abundance and grazing impact on primary producers [41,42]. A second major stressor of interest in many freshwater systems is re-oligotrophication, which often follows periods of eutrophication [43–45]. Although it has proven to be a key measure for the restoration of eutrophied lakes, re-oligotrophication is likely to lead to considerable nutrient stress in consumers [43]. Nutrient deficiency in oligotrophic systems, combined with warming and increased CO_2_, jointly influence primary production, and contribute to an increased stoichiometric mismatch between phytoplankton and their herbivore consumers [46,47]. These stoichiometric changes further constrain performance of consumers through either nutrient shortage [48–51] or excess carbon [52,53]. The combined effects of different stressors, such as increased salinization and nutrient deficiency, may lead to strong declines in biodiversity and ecosystem functioning [54–56].

Natural populations adapted to either increased salinity or nutrient limitation may increasingly face challenges from the other stressor. For example, oligotrophic lakes may be subject to anthropogenic salinization such as the application of road salts [57], whereas oligotrophication may also occur in salinized systems. For many systems, it remains unclear how the recent evolutionary history of consumers will determine their response to novel environmental changes. Studies have documented adaptive evolutionary responses of freshwater organisms to either increased salinity [58–60] or nutrient limitation [61–63]. Adaptation to increased salinity is generally observed to involve processes related to iono- and osmoregulation [59,64]. Adaptation to oligotrophic conditions is less well understood mechanistically but is likely to result in various changes in the elemental phenotype [65–67]. It remains unclear how stress due to salinity and nutrient limitation may interact, and how adaptive microevolution to either salinization or nutrient limitation would affect the ability of freshwater organisms in tolerating the other.

To investigate how adaptation to nutrient limitation or increased salinity may affect the ability of organisms to cope with the other, and whether the sequence of exposure matters, we used clonal lines (genotypes) of populations of the rotifer *Brachionus calyciflorus* s.s. from former selection experiments during which populations had adapted to either phosphorus-limited food (LP) [63] or saline conditions (HS) [29]. Using a common garden design, we quantified the change in the population growth rate of the LP and HS-selected lines when moved from a benign environment to P-limited food and salt stress conditions.

## 2. Methods

### (a) Rotifer and algae cultures

The rotifer *B. calyciflorus* is a cyclical parthenogenetic monogonont zooplankter and is an ideal model organism to study microevolution because of their short generation time and ease of manipulation [68]. Prior to this experiment, stock cultures of *B. calyciflorus* were maintained at room temperature under continuous light conditions fed with P-sufficient food (Na^+^: 0.0005 g l^−1^, Cl^−^: 0.018 g l^−1^) on a daily basis. We used the green algae *Chlamydomonas reinhardtii* as a food resource for rotifers. Algae were cultured in 2 l-chemostats under two nutrient regimes, i.e. P sufficient (algal molar C : P = 120, ‘HP’) and P deficient growth conditions (algal molar C : P = 650, ‘LP’). We monitored cell diameter and cell density of the algae during the course of the experiment and measured the carbon (C), phosphorus (P) and nitrogen (N) content of the algae at the start of the experiment (for more details, see electronic supplementary material).

### (b) Previous evolution experiments

We used clones derived from two separate experimental evolution studies, which were adapted to either high salinity conditions using sodium chloride (NaCl) or phosphorus-limited algal food sources [29,63]. Both evolution experiments were initiated using ‘ancestral’ populations composed of genotypes hatched from diapausing eggs isolated from the surficial sediments of seven freshwater ponds in the Netherlands. Clones were maintained in batch cultures since hatching with P sufficient *C. reinhardtii* in standardized laboratory conditions.

The salt selection experiment [29] was started by creating three HP-fed ancestral populations with identical genetic composition (44 genotypes). Starting at a salinity level of 2 g l^−1^, populations were allowed to grow until high population density triggered sexual reproduction and the formation of dormant propagules, which were collected and hatched to establish new clonal lines. These clonal lines were subsequently combined to reconstitute the populations, which were then exposed to a higher salinity level (3 and then 4 g l^−1^). This evolution experiment lasted for 82 days. Common garden experiments subsequently demonstrated the adaptation of clonal lines to salt compared to populations that had not been selected under salt (for more details, see electronic supplementary material) [29].

The selection experiment in low-phosphorus conditions was initiated by exposing seven replicate multiclonal ancestral populations to a LP food treatment. Each initial population consisted of two individuals of the same set of 30 different genotypes. From the second day onwards, a subset of 60 haphazardly selected individuals and all the resting eggs were transferred to a fresh food suspension every 24 h. This process was selected for genotypes with high intrinsic growth rates during a period of 36 days. A common garden experiment subsequently demonstrated that the LP-selected populations had higher population growth rates with LP resources than both ancestral and control populations selected with high P resources (for more details, see electronic supplementary material) [63].

### (c) Common garden experiment

We conducted a common garden experiment to investigate how adaptation to either P limitation or increased salinization affects rotifer growth when confronting the alternate stressor. For this experiment, we used genotypes (clones) from the salt-adapted, LP-adapted and ancestral populations. For the common garden experiment, we used three randomly selected genotypes from each evolution history.

The common garden experiment originally consisted of 108 experimental units, i.e. 2 food quality treatments (HP and LP food) × 2 salinity treatments (HS and NS) × 3 selection histories (ancestral, salt-adapted and LP-adapted) × 3 clones × 3 replicates per clone, each fed with algae from a different chemostat. Initially, the HS treatments received NaCl addition at a concentration of 3 g l^−1^, the same concentration as the salt-adapted populations had been selected for [29]. This concentration of salt corresponds to conditions observed in natural freshwater ecosystems due to, for example, the use of road salts in winter [69]. Unfortunately, the combined effects of 3 g l^−1^ salt and LP food resulted in a rapid decline of populations that lacked prior adaptation to salt. We therefore repeated the experiment by applying 3 g l^−1^ NaCl (further also referred to as ‘high’ salinity, HS) in the HP and 1.5 g l^−1^ NaCl (further referred to as ‘moderate’ salinity, MS) in the LP food treatment.

We started each experimental population by transferring 10 haphazardly selected individuals from clonally growing stock cultures to 16 ml well plates filled with 8 ml media which contained the corresponding stressor treatment. All algae (LP or HP) were provided at a density of 1000 µmol C l^−1^. The plates were incubated at 23°C ± 1°C under continuous darkness. Every 24 h, wells were checked under a stereomicroscope and the number of females was counted. At this time, 10 individuals from each unit were haphazardly selected and transferred to a new well with fresh food suspension and the same experimental treatment. We continued the experimental process until the population growth rate for each experimental unit stabilized, as evidenced by the absence of significant temporal trends (electronic supplementary material, table S1). Upon achieving this steady state (from day 7 onwards), we selected data from the last 3 days of the experiment (days 8, 9 and 10) for our formal data analysis.

### (d) Data analysis

For the final 3 days of the experiment, rotifer population growth rates were calculated for each experimental unit as (ln*N*_*t*_ − ln*N*_0_)/*t*, where *N*_0_ and *N*_*t*_ represent the population size at the start and end of each 24 h period, and where *t* is the time interval between the two counts (1 day). Subsequent analyses made use of the mean of these three values.

We first investigated the effect of the common garden treatments on population growth rates. Differences in salinity between HP–HS and LP–MS treatments precluded the statistical evaluation of a three-way interaction. We therefore performed two analyses, i.e. one per food quality treatment (HP and LP). For each of these treatments, we employed linear mixed effects models with adaptation history and salinity as fixed factors, and clone and food source (i.e. chemostat replicates) as random factors by design.

To investigate how adaptation to one stressor may influence the capacity of rotifers to tolerate another stressor, we compared growth rate reductions of adapted populations with those of ancestral populations when going from benign to stressful environments. For example, to assess the impacts of LP adaptation on the ability of rotifers to cope with increased salinity, we calculated the growth rate reduction of both LP-adapted and ancestral populations going from NS to HS treatments (△*r*_sal_ = △*r*_HS_ − △*r*_NS_), at the level of each clone and chemostat replicate. Evolved cross inhibition was inferred from a dependent *t*‐test if the ^△^*r*_sal_ values of LP-adapted populations were significantly lower than the ancestral populations. Conversely, evolved cross-tolerance was inferred if the △*r*_sal_ values of the LP-adapted populations were significantly greater than the ancestral populations. This test was performed separately for populations fed HP food and for those fed LP food. Similarly, the impacts of salt adaptation on the ability of rotifers to cope with food P limitation were assessed by testing for differences in the reduction in population growth rate when going from HP to LP treatments (△*r*_P_ = △*r*_LP_ − △*r*_HP_) between ancestral and salt-adapted populations. However, this could only be calculated in the NS treatments because of the difference in salinity levels between HP and LP treatments. To control for the risk of Type I errors arising from multiple comparisons, we applied the Holm–Bonferroni method ensuring that the family-wise error rate was controlled at *α* = 0.05 based on all independent *t*-tests performed in the study [70].

Linear mixed models and one-way ANOVA were applied to test the responses of algal size and carbon content to different nutrients, respectively, incorporating time and chemostat replicates as random factors (for more details, see electronic supplementary material). All statistical analyses were performed in the R software environment v. 3.4.1 [71], and the linear mixed models were performed using the ‘lmer()’ function in the *lme4* package [72]. The Type III ANOVA tests on the linear mixed models were conducted using the ‘anova()’ function from the ‘lmerTest’ package [73], where we employed Satterthwaite’s approximation for degrees of freedom. The post hoc comparisons were conducted using the ‘emmeans’ package, with Holm’s method applied to adjust for multiple comparisons and control for the family-wise error rate at *α* = 0.05.

## 3. Results

**Figure 1.**
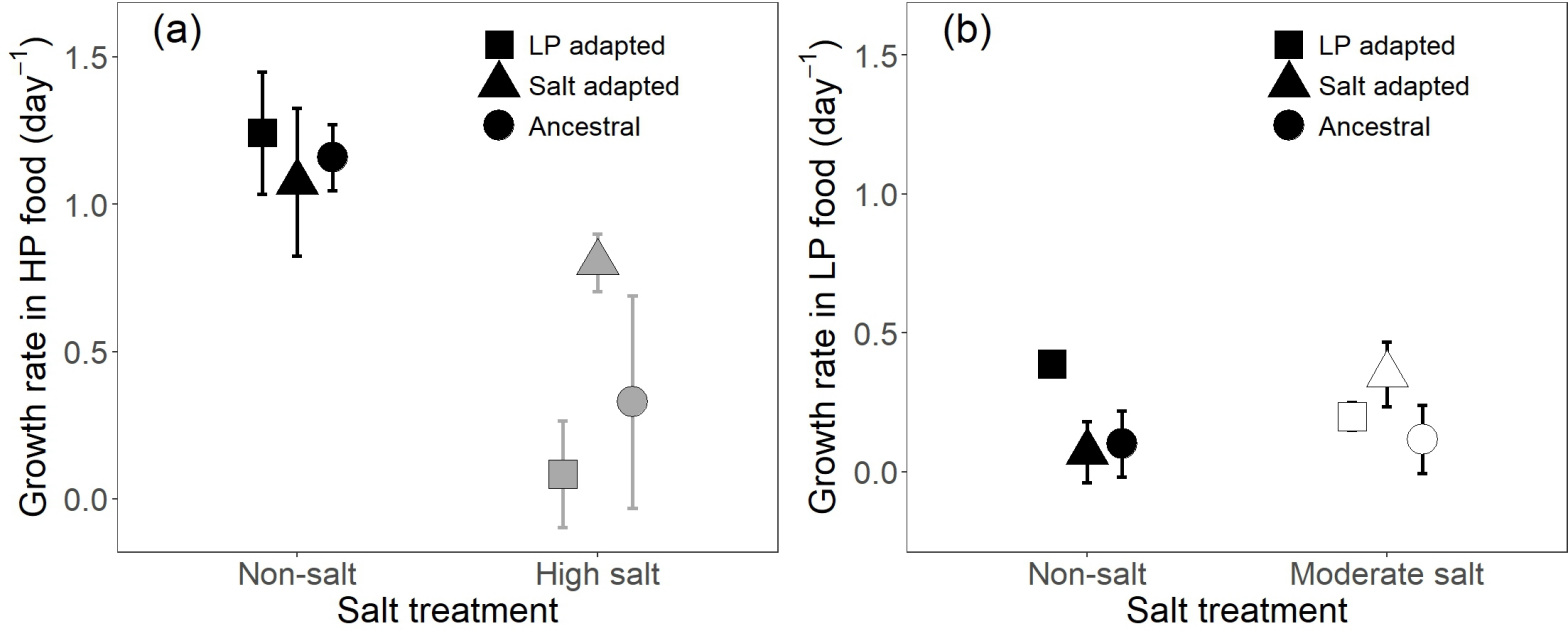
Population growth rates of rotifers in response to different combinations of stressors. (a) HP food and (b) LP food. Symbol values with error bars refer to means of the three replicate clones ±2 × s.e. Note that salt concentrations in the salt treatment were higher (3 g l^−1^ NaCl; denoted as dark grey symbols) in (a), whereas they were moderate (1.5 g l^−1^ NaCl; denoted as empty symbols) in (b).

Rotifer growth rates were generally lower when exposed to LP compared with HP food (figure 1). When provided HP food, the effect of salinity depended on adaptation history (figure 1a; table 1). Although population growth for all adaptation histories was significantly lower in the high compared to no salt treatment, the magnitude of the effect differed by adaptation history (Holm post hoc: ancestral populations: mean difference = 0.829 (day^−1^) ± 0.058 s.e., *p* < 0.001, LP-adapted populations: mean difference = 1.157 (day^−1^) ± 0.058 s.e., *p* < 0.001, salt-adapted populations: mean difference = 0.273 (day^−1^) ± 0.058 s.e., *p* < 0.001; figure 1a; electronic supplementary material, table S2). As a result, at high salt concentrations, the population growth rate of salt-adapted populations was greater than LP-adapted (Holm post hoc mean difference = 0.717 (day^−1^) ± 0.139 s.e., *p* = 0.01; electronic supplementary material, table S2) and marginally higher than ancestral (Holm post hoc mean difference = 0.472 (day^−1^) ± 0.139 s.e., *p* = 0.077; electronic supplementary material, table S2) populations.

**Table 1.** Analysis of variance of the linear mixed models for HP and LP food, respectively, for which salinity (SAL) and population selection history (SH) were specified as fixed factors, and clones were identified as random factors. SS: sum of squares, MS: mean squares, NumDF: degrees of freedom.

variables	SS	MS	NumDF	*F*-value	*p*
population growth rate in HP food					
SAL	7.65	7.65	1	509.7	<0.001
SH	0.07	0.03	2	2.27	0.18
SAL × SH	1.80	0.9	2	59.83	<0.001
population growth rate in LP food					
SAL	0.017	0.017	1	2.00	0.16
SH	0.08	0.04	2	4.87	0.051
SAL × SH	0.49	0.25	2	29.06	<0.001

In LP food, the effect of salinity again strongly depended on adaptation history (figure 1b; table 1). The population growth rates under moderate compared with non-saline conditions were significantly lower for LP-adapted populations (Holm post hoc: mean difference = 0.189 day^−^¹ ± 0.043 s.e., *p* < 0.001; figure 1b; electronic supplementary material, table S3) and significantly higher for salt-adapted populations (Holm post hoc: mean difference = 0.279 day^−^¹ ± 0.043 s.e., *p* < 0.001; figure 1b; electronic supplementary material, table S3). In contrast, no significant change was observed for the ancestral populations (mean difference = 0.017 day^−^¹ ± 0.043 s.e., *p* = 1; figure 1b; electronic supplementary material, table S3). In the non-saline environment, LP-adapted populations had significantly higher population growth rates than salt-adapted and ancestral populations (LP-adapted versus salt-adapted: mean difference = 0.317 (day^−1^) ± 0.067 s.e., *p* = 0.012; LP-adapted versus ancestral: mean difference = 0.288 (day^−1^) ± 0.067 s.e., *p* = 0.021; figure 1b; electronic supplementary material, figure S1 and table S3). However, in the moderately saline environment, population growth rates did not differ between the LP-adapted and the other adaptation histories (Holm post hoc: LP-adapted versus salt-adapted: mean difference = 0.151 (day^−1^) ± 0.067 s.e., *p* = 0.382; LP-adapted versus ancestral: mean difference = 0.082 (day^−1^) ± 0.067 s.e., *p* = 1; figure 1b; electronic supplementary material, figure S1 and table S3). Population growth rates of salt-adapted populations in the treatment combining non-saline conditions and LP food did not differ from ancestral populations (Holm post hoc: mean difference = 0.029 (day^−1^) ± 0.067 s.e., *p* = 1; figure 1b). However, under moderately saline conditions, salt-adapted populations had higher population growth rates than ancestral populations (Holm post hoc: mean difference = 0.233 (day^−1^) ± 0.067 s.e., *p* = 0.056; figure 1b).

**Figure 2.**
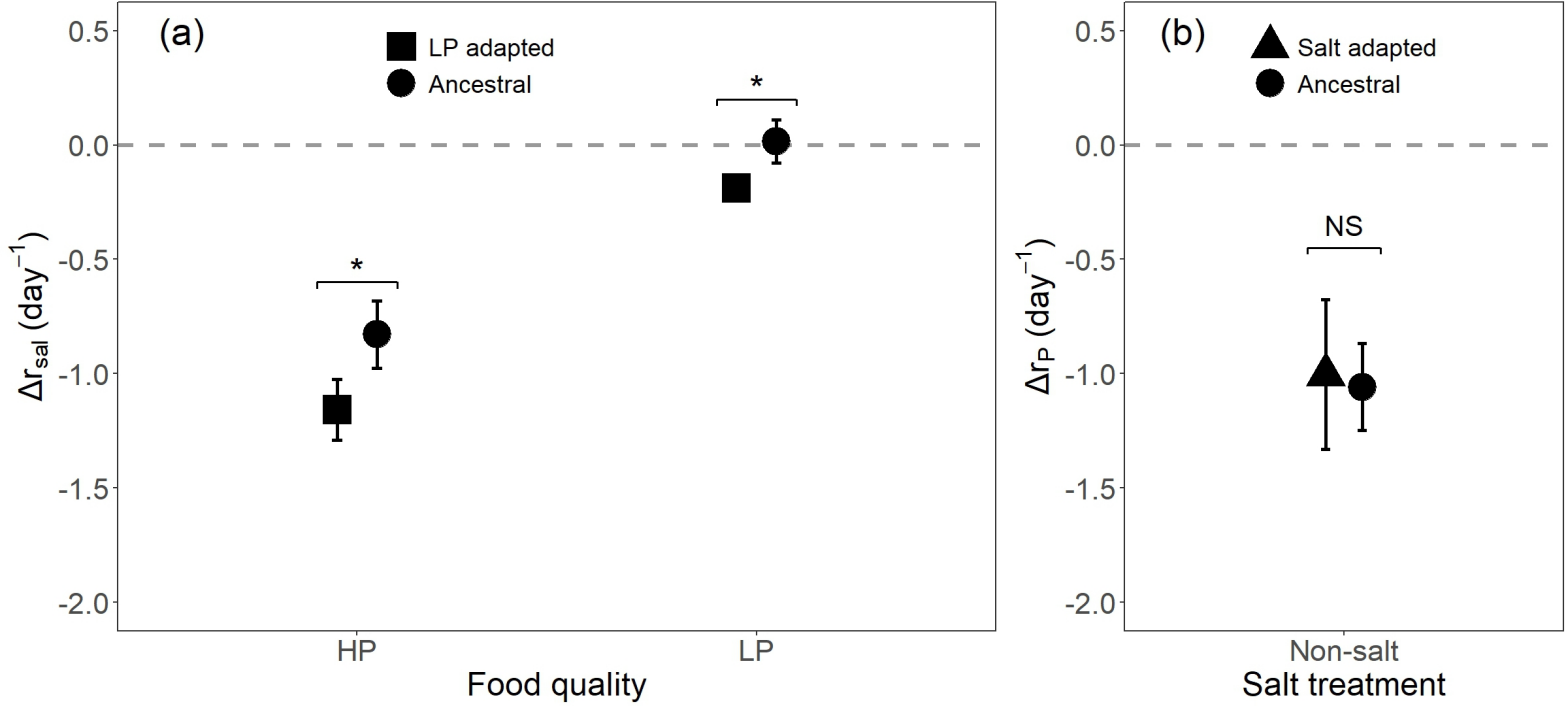
Growth rate responses (△r) to contrasting experimental treatments. (a) growth rate reduction (△$r_{sal}$) of LP-adapted and ancestral populations caused by increased salinity (i.e. from 0 to 3 g l^−1^ NaCl in HP and from 0 to 1.5 g l^−1^ NaCl in LP), and (b) growth rate reduction (△$r_{P}$) of salt-adapted and ancestral populations caused by food P limitation (i.e. from HP to LP food). Black squares, triangles and circles represent △r values of LP-adapted, salt-adapted and ancestral populations, respectively. Dashed lines at 0 value indicate the absence of treatment effects. Symbol values and error bars represent the mean growth rate reduction across three replicate clones ± 2 × s.e. Asterisks indicate the level of statistical significance: *p < 0.05; n.s.: not significant.

According to Δ*r*_sal_ values, the negative effect of increased salinity was consistently stronger for LP-adapted populations than ancestral populations in both HP (paired *t*‐test: *t*_8_ = 3.29, *p*_adjusted_ = 0.01, mean difference = −0.33) and LP food (paired *t*‐test: *t*_8_ = 3.36, *p*_adjusted_ = 0.03, mean difference = −0.21; figure 2a), demonstrating the existence of evolved cross inhibition. When going from HP to LP food, we observed no difference in the reduction in population growth rate between salt-adapted and ancestral populations under non-saline conditions (paired *t*‐test: *t*_16_ = −0.40, *p*_adjusted_ = 0.697, mean difference = 0.05; figure 2b).

The diameter (*F*_1,4_ = 70.4, *p* = 0.001; electronic supplementary material, figures S2 and S3a) and carbon content (*F*_1,4_ = 8.315, *p* = 0.045; electronic supplementary material, figures S2 and S3b) of an algal cell were both significantly greater in the LP compared with HP conditions.

## 4. Discussion

Understanding and predicting the outcomes of anthropogenic changes requires consideration of multiple stressors [6,8]. Evidence for rapid adaptation highlights the critical role of microevolutionary history in assessing stressor effects [14,74,75]. In the presence of multiple stressors, evolutionary outcomes can be complex, influenced not only by stressor type and number [7,76] but also by their sequence and temporal dynamics [77]. Our findings demonstrate that microevolutionary adaptation to one stressor can significantly affect a population’s ability to cope with others. Notably, we observed non-reciprocal effects: while adaptation to P-deficient food reduced tolerance to salt, adaptation to salt did not impair tolerance to P-deficient food.

Adaptation to P-limited conditions reduced the salinity tolerance of rotifer populations. The reductions in growth rate due to high salinity were more pronounced in the LP-adapted populations compared to the ancestral population, regardless of whether they were provided with HP or LP algae (figure 2). This demonstrates that adaptation to LP resources induces cross-inhibition under salt stress. The mechanisms underlying such cross-inhibition remain largely unknown. Salt stress requires high metabolic energy to power ion transport [78], and if P-limitation adaptation reallocates P away from molecules critical for energy metabolism, such as phosphagens, this could impair stress responses [79]. Cell membrane lipid composition, including phospholipids, also plays a critical role in maintaining ion transport and osmoregulatory capacity. Reduced phospholipid levels in membranes [80] is another way of how P-limitation may compromise salt tolerance. Alternatively, selection for the enhanced elimination of C excess under stoichiometric imbalance may prove maladaptive when salt stress increases energy demands, though data by Lemmen *et al.* [63] are not providing much support for this idea.

Previous studies on rotifer adaptation to P-limited resources found no evidence for trade-offs between performance under HP and LP food conditions [62,63], though these observations were under benign conditions. The current finding that LP adaptation incurs costs under salt stress suggests that trade-offs may become more evident under stressful conditions [23,81]. This aligns with broader literature, where adaptation to specific stressors often results in reduced tolerance to others [17–19,82].

In contrast, we found no evidence for cross-inhibitory effects of adaptation to salt stress regarding population responses to food P-limitation. LP food strongly reduced population growth rates of all populations irrespective of population selection history. In non-saline conditions, growth rate reductions due to LP food were similar between ancestral and salt-adapted clones (figures 1b and 2b). We were not able to compare responses of these populations to LP food in a saline environment due to the limitations of our experimental design. Nevertheless, the reaction norms of the populations revealed some intriguing responses. Under LP conditions, moderate salt conditions did not appear to have a compounding negative effect on growth rate. Apart from a reduction in population growth of LP-adapted populations, we found no significant difference between the non-salt and moderate salt treatments for ancestral populations in the LP food treatment. Remarkably, salt-adapted populations showed an increase in population growth in the moderate salt treatment combined with LP food. On initial consideration, this could be interpreted as a cost of adaptation, where enhanced performance under saline conditions trade-off with reduced performance in non-saline conditions, consistent with a pattern of local adaptation. However, the observation that growth of these populations under non-saline conditions is not lower than that of ancestral populations does not support this interpretation. Similarly, Zhao *et al.* [29] found no evidence for a cost of salt adaptation under freshwater conditions. An interesting question is then how the increased growth of salt-adapted populations in a moderately saline environment can be explained. At first glance, it may seem to reflect their ability to thrive in conditions to which they have adapted. However, salt is a stressor, and one might expect adaptation to such a stressor to result in a reduced growth penalty compared with non-adapted populations, rather than a relative growth increase.

An intriguing alternative hypothesis is that the higher population growth rates of the salt-adapted populations in the moderate saline LP environment compared with the non-saline LP environment reflects an evolved cross-tolerance to LP food. Physical and chemical stressors often impair organism performance by disrupting energy metabolism [78,83,84], primarily due to the energetic demands of maintaining homeostasis and activating molecular protection and repair mechanisms [85,86]. LP food typically contains an excess of carbon [52,53], as in our case, the algae constituting the LP diet exhibited higher cell volumes, carbon content per cell and molar C : P ratios compared with those in the HP diet. Adaptation to salt may have involved the upregulation of energy-intensive mechanisms, such as iono- and osmoregulation [59,64,87,88]. While the excess carbon in LP food is generally considered a factor that can negatively impact zooplankton growth [89], the increased energy demands of salt-adapted populations in a moderately saline environment may have allowed them to better tolerate the adverse effects of this surplus carbon in their diet [52,53].

Our study enhances our knowledge on how organisms' responses to multiple stressors are shaped by their recent evolutionary history [4,28]. A growing body of research underscores the intricate role of evolutionary history in shaping responses to environmental stressors (e.g. [90,91]), revealing patterns of either evolved cross-tolerance [26,29] or evolved cross-inhibition [16–19]. Our study contributes a novel perspective by highlighting the non-reciprocity of adaptive outcomes. Specifically, while adaptation to low-phosphorus food reduced rotifer tolerance to salinity, adaptation to salinity did not diminish tolerance to phosphorus-deficient food. This finding underscores the importance of considering the sequence of adaptation when studying responses to multiple stressors. A critical next step would be to explore how adaptation to one stressor influences the microevolutionary potential of populations [92] to subsequent stressors, and how this is shaped by the specific order of stressor regimes. Overall, our work advocates for a comprehensive and in-depth exploration of the impacts of historical selection regimes on the tolerance, persistence and adaptive capacity of populations in a variable, multi-stressor world.

Our study also holds potentially important implications for natural ecosystems. Salinization [32,93,94] and changes in nutrient availability, including both oligotrophication [45,95–97] and eutrophication [98–100], are increasingly threatening the biodiversity and functionality of freshwater and terrestrial ecosystems. Our findings reveal that populations adapted to low-phosphorus conditions exhibit reduced tolerance to additional stressors, such as salinity, compared with their ancestral populations. This indicates that the vulnerability of populations to emerging stressors may depend on the trophic status of their habitat. Notably, the salt concentrations used in our experiments are within the range of natural salinities reported in both lake [101,102] and stream [103] ecosystems. Our results imply that salt pollution could have a disproportionately detrimental impact on organisms in oligotrophic systems compared to eutrophic ones, suggesting a heightened vulnerability of nutrient-poor aquatic environments to rising salinity levels.

In contrast, salt-adapted populations exhibited reduced performance when switching from moderate saline to non-saline conditions in LP environment, indicating that reduction in salinity levels within moderately saline, oligotrophic freshwater systems may compromise the performance of locally adapted populations. While a more comprehensive understanding of such effects is still needed, such knowledge will probably be relevant to managers, who need to recognize that prior adaptations to stressors can significantly influence an organism’s response to subsequent environmental changes. Furthermore, the sequence and duration of management interventions can lead to varied outcomes, underscoring the complexity of ecosystem responses. In this context, we advocate for future research to take an additional step by conducting sequential evolution experiments. Such studies could systematically explore microevolutionary adaptations to sequentially imposed stressors over successive generations, providing valuable insights into the dynamics of adaptation under complex environmental scenarios.

## 5. Conclusion

The interplay of multiple stressors is widely recognized as a key determinant of the effects of anthropogenic stressors on organisms in natural systems [1,8,104]. However, the role of microevolutionary adaptation in a multi-stressor context has only recently gained attention as a critical area of study [28,77]. Our study represents a significant step forward by highlighting the importance of the history of selection regimes and the specific sequence of stressors faced by populations. Overall, this research underscores the need to expand the scope of studies on microevolutionary histories and their implications for organismal responses to stressors.

## Data Availability

All annotated code and data are available at [105]. Supplementary material is available online [106].
